# Gas Transport Phenomena and Polymer Dynamics in PHB/PLA Blend Films as Potential Packaging Materials

**DOI:** 10.3390/polym12030647

**Published:** 2020-03-12

**Authors:** Valentina Siracusa, Svetlana Karpova, Anatoliy Olkhov, Anna Zhulkina, Regina Kosenko, Alexey Iordanskii

**Affiliations:** 1 Department of Chemical Science (DSC), University of Catania, Viale A. Doria 6, 95125 Catania, Italy; 2 Plekhanov Russian University of Economics, Stremyanny per. 36, 117997 Moscow, Russian Federation; karpova@sky.chph.ras.ru (S.K.); aolkhov72@yandex.ru (A.O.); 3 Semenov Institute of Chemical Physics, Kosygin str. 4, 119991 Moscow, Russian Federation; annazhulkina@gmail.com (A.Z.); vadim-parfenov5@rambler.ru (R.K.); aljordan08@gmail.com (A.I.)

**Keywords:** poly(3-hydroxybutyrate) (PHB), polylactic acid (PLA), biomaterials, gas permeability, gas diffusion, segmental dynamics, electron spin resonance (ESR), scanning electron microscopy (SEM), differential scanning calorimetry (DSC)

## Abstract

Actually, in order to replace traditional fossil-based polymers, many efforts are devoted to the design and development of new and high-performance bioplastics materials. Poly(hydroxy alkanoates) (PHA_S_) as well as polylactides are the main candidates as naturally derived polymers. The intention of the present study is to manufacture fully bio-based blends based on two polyesters: poly (3-hydroxybutyrate) (PHB) and polylactic acid (PLA) as real competitors that could be used to replace petrol polymers in packaging industry. Blends in the shape of films have been prepared by chloroform solvent cast solution methodology, at different PHB/PLA ratios: 1/0, 1/9, 3/7, 5/5, 0/1. A series of dynamic explorations have been performed in order to characterize them from a different point of view. Gas permeability to N_2_, O_2_, and CO_2_ gases and probe (TEMPO) electron spin resonance (ESR) analyses were performed. Blend surface morphology has been evaluated by Scanning Electron Microscopy (SEM) while their thermal behavior was analyzed by Differential Scanning Calorimetry (DSC) technique. Special attention was devoted to color and transparency estimation. Both probe rotation mobility and N_2_, O_2_, and CO_2_ permeation have monotonically decreased during the transition from PLA to PHB, for all contents of bio-blends, namely because of transferring from PLA with lower crystallinity to PHB with a higher one. Consequently, the role of the crystallinity was elucidated. The temperature dependences for CO_2_ permeability and diffusivity as well as for probe correlation time allowed the authors to evaluate the activation energy of both processes. The values of gas transport energy activation and TEMPO rotation mobility are substantially close to each other, which should testify that polymer segmental mobility determines the gas permeability modality.

## 1. Introduction

The biodegradable biopolyester family comprising polylactides (PLA)s and polyhydroxyalcanoates (PHA)s has to be considered as an attractive opportunity to the family of conventional petrol-based polymers. Owing to their specific transport behavior [1,2,3], appropriate mechanical properties [4,5], enhanced functionality [6,7], as well as in virtue of controlled biodegradability [8], both biopolymer categories are designated to replace the synthetic plastics in innovative areas such as biomedicine, environmental improving, and especially the packaging industry [9,10]. The high upsurge in human population and hence the corresponding food product expenditure enlarge exogenous pollutions, as well as a waste cluster area. The extremely important impact on enhancing the anthropogenic pollution belongs to the short-term plastic packaging such as a custom care and one-off household utensils. Therefore, PLAs and PHAs elaboration as novel packaging materials could minimize the persistent harmful wastes in aquatic and terrestrial environments [11,12].

Poly(3-hydroxybutyrate) (PHB) is the principal and a most widely used member of the PHAs family that demonstrates the higher potentiality for replace fossil-based synthetic packaging [13]. In homopolymeric status, PHB shows a high stereo-regularity, leading to an extremely large crystallinity up to 70–75 wt.%. The optimal combination of biopolymer crystalline entities (crystallites, lamellae, spherulites) and its intercrystalline transitional chains promote favorable mechanical properties such as high elasticity modulus of around 2.5–3 GPa and tensile strength at break of 35–40 MPa [14]. Thanks to its structure, this biopolymer displays thermophysical and mechanical characteristics similar to polystyrene and isotactic polypropylene [15]. The extensional lamellae and spherulyte morphology, furthermore, constitute a good barrier structure with appropriate low permeability values for atmospheric gaseous components [13,16,17] and water vapor [13,14,18,19]. 

As well reported in literature [1,20,21,22,23], having molecular permeability and diffusivity dependent on biopolymer morphology, their knowledge is identified as crucial factors for determining the inherent packaging behavior, including shelf life terms, food saving, and antibacterial resistance. 

The PLA family, with polymers of various proportion in D-lactic and L-lactic stereoisomers, dominates on the biodegradable polymers’ market due to their appropriate characteristics such as processability, high transparency, rigidity, and relatively low cost (today it is about 2.5 $/kg). Nonetheless, along with tailoring characteristics like traditional petro-based thermoplastics such as iso-propylene (i-PP), polystyrene (PS), and poly(ethyleneterphtalate) (PET), its high glass transition temperature (T_g_) provides a more redundant brittleness, impairing barrier behavior for packaging. Beyond the above positive attributes, PLA is characterized by a number of noticeable flaws that confines its implementation as constructional and packaging materials chiefly. According to the barrier characteristics against gas, water, and low-molecular organic substances, PLA is evaluated as packaging with intermediate permeability coefficient values between PS and PET [24]. Actually, the inherent confinements include the low-speed crystallization, high values of T_g_ leading to extra brittleness, and low toughness. Furthermore, via electrospray ionization mass spectrometry, researchers such as Bor, Alin, and Hakkarainen [25] have discovered lactide acid oligomers release from PLA films into the fat food mimetic media (96% ethanol solution), which may spoil the food quality to a certain extent. 

To avoid the characteristic disadvantages of PHB and PLA, there are several updating approaches that include copolymerization [26], physical action on crystallization via nucleation agents loading or/and annealing [27,28], plasticization [29,30], surface modification [31], filler bulk embedding [32], and so on. The most common technique to tailor relevant packaging features of the biopolyesters includes their blending under appropriate conditions through melt or co-solution procedures [30,33,34,35].

Using the similarity of molecular structure for two biopolyesters, PHB/PLA blends and composites have been prepared and carefully investigated [23,29,34,36,37,38,39,40]. One of the principal motivations for creating PHB/PLA compositions is to obtain new packaging materials that are free from the above homopolymers disadvantages and especially in such areas as controlled transport, tailored mechanical behavior, and operated biodegradability in post-exploitation landfill conditions.

At the molecular level, both biopolyesters with analogous terminal (-ОН, -СООН) and polymer backbone (-С-О(С=О)-С) groups are conducive to the interaction that reflects trans-esterification reactions [41]. Enabling the conditions for the reactions of that type contributes to solid-state polymerization [42] and reactive compatibility between PHB and PLA using spacers [34,43]. However, the new polymeric adduct, where the macromolecules are chemically connected, is transformed into novel matrix with diffusional, dynamic, and mechanical properties, which are essentially different from the initial homopolymers. On the other hand, the additives like plasticizers and compatibilizers e.g., from low-molecular acetyl tri-n-butyl citrate [44] to oligomers [45], lead to compatibility improvement as well. In the elaboration of polymer packaging, the plasticizer migration, desorption, and leaching [45,46] should be carefully assessed. In the framework of this publication, the developing work of Yeo et al. [27] is of great interest. Here, by structural modification, the mechanical features of PHB/PLA composition have been dramatically improved. Authors elaborated the PHB-based filler particles with effective nucleation activity and extremely high toughening of PLA matrix. 

While numerous publications were devoted to the mechanical behavior of the PHB/PLA blend films [47,48,49], their gas transport characteristics have been represented relatively poorly. The literature mainly displays the data on homopolymer gas permeability, predominantly with oxygen and carbon dioxide, and water vapor permeability [1,13,17,19,38,50,51,52], as also reported by authors of this paper on PHB [53] and PLA [31] homopolymers, also describing the corresponding data on segmental mobility (obtained by electron spin resonance (ESR) technique), under the assumption that the chain mobility controls the gas diffusion mechanism in bio-polymers. 

In this paper, expanded feasibility studies were performed for permeability as well as gas diffusivity and solubility, namely the assessment of features that determine O_2_, N_2_, and CO_2_ transfer. These findings, in combination with the structure-morphology pattern, could be interpreted as ground barrier packaging characteristics, which are necessary for a cohesive interpretation of more elaborated packaging with active functions such as antimicrobial performance and food-modifier controlled release.

## 2. Materials and Methods 

PHB and PLA are the most common bio-based polymers. Their biodegradable behavior is well utilized to solve waste and plastic pollution environmental concerns. As a consequence, in order to develop and extend their applications as blends, it is important to understand their properties related to their chemistry.

### 2.1. Materials

PHB was kindly supplied by Biomer Co (Krailing, Germany), personally by Dr. Urs Haenggi. Initially, the biopolymer was presented as white powder with particle size of 3–7 μm, MW = 2.06 × 10^5^ Da. PLA as micropellets (MW = 120,000) was purchased from Nature Works, LLC (Minnetonka, MN, USA), product 3801X, with > 1% of d-isomer. All materials were used as received, without any further purification.

### 2.2. Biopolymers Film Preparation

PHB and PLA films were prepared by solvent casting procedure. PHB powder and PLA pellets were weighed, dissolved in chloroform (7% wt/v), and heated on a hot plate with magnetic stirrer (Heidolph Instruments GmbH & CO. KG, Schwabach, Germany), under vigorous stirring at 70 °C (±0.1 °C), for 12 h, until complete dissolution. The obtained solutions were cooled at room temperature and, subsequently, 11 ± 0.5 ml were plated in glass petri-dishes (7 cm diameter) and allowed to slow evaporation (for 48 h) at room temperature (25 ± 1°C) and 60 ± 2% of relative humidity. To ensure a complete solvent removal, the obtained films were dried under vacuum for 48 h (instrument used), in order to reach a constant weight. The dried films were detached from the petri-plates carefully by peeling and then stored at least 2 days at 40 °C temperature, to remove the effect of residual structural relaxation, before characterization.

For PHB/PLA blends, a total concentration of 7% wt/v was dissolved in chloroform, under vigorous stirring at 7 °C, for 12 h. Blends of PHB/PLA with the following weight ratio were obtained: 1/0 (PHB), 9/1, 7/3, 5/5, and 0/1 (PLA). All films were allowed to dry before characterization. Films were stored in a desiccator (with silica gel) prior to testing other parameters. 

### 2.3. Scanning Electron Microscopy

The surface morphology of the PHB/PLA films was studied by SEM technique with the JSM-6510LV JEOL LLC scanning electron microscope (Tokyo, Japan) on samples coated with vapor-deposited gold (Au). The samples were mounted onto Al stud and coated with Au using a sputter (Polaron E5200, Denton Vacuum, Moorestown, NJ, USA), set at 25 mA, for 10 s. 

### 2.4. Differential Scanning Calorimetry (DSC) Measurements

Differential scanning calorimetry (DSC) tests, to evaluate the characteristic temperatures and the corresponding phase change enthalpies, were carried out on a DSC Q-20 instrument (TA Instruments, New Castle, De, USA) in a nitrogen atmosphere at a heating rate of 10 °C/min, equipped with a liquid sub-ambient accessory and calibrated with high purity standards. Polymer films were cut into small pieces of about 2 mm^2^ and 10–12 mg in weight and placed in a 50 μL sealed aluminum pan. After an isotherm of 5 min at 20 °C, samples were heated with a scanning rate of 10 °C/min, from 20 to 180 °C (first scan) and then, after a further isotherm of 2 min at 180 °C, were cooled to 20 °C at a rate of 10 °C/min. Finally, after an isotherm of 3 min, samples were reheated from −20 °C/min to 180 °C/min at 10 °C/min (second scan). All the experiments were performed under nitrogen flow (20 cm^3^/min). The melting temperature (T_m_) was determined as the peak value of the endothermic phenomena in the DSC curve. The melting enthalpy (∆Hm) of the crystal phase was calculated from the area of the DSC endothermic peak. T_m_ and ∆H_m_ values were collected from the first scan. The average error in thermal effect measurements was approximately ±3%. 

### 2.5. ESR Characterization

The X-band ESR spectra were recorded on an EPR automated spectrometer (EPR-B Instrument producted in ICPRAS, Moscow, RF). The microwave power in the resonator did not exceed 7 mW, in order to avoid signal saturation effects. When recording the spectra, the modulation amplitude was always substantially smaller than the width of the resonance line and did not exceed 0.5 G. The probe was a stable nitroxyl radical TEMPO synthesized in Emanuel Biochemical Physics Institute, RAS. The radical was introduced into the films from the vapor phase at a temperature of 60 °C. The concentration of the radical in the polymer did not exceed 10^–3^ mol/L. The experimental spectra of the spin probe in the region of slow motion (the correlation time of probe rotation τ > 10^–9^ s) was analyzed within the framework of the isotropic Brownian rotation model using the program described in [54]. For modeling the spectra, we used the following main values of the *g* tensor and the hyperfine radical interaction tensor: *g_xx_* =2.0096, *g_yy_* = 2.0066, *g_zz_* = 2.0025, *A_xx_* = 7.0 G, *A_yy_* =5.0 G, *A_zz_* = 35.0 G. Note that the value of *Azz* was determined experimentally from the EPR spectra of the nitroxyl radical in the polymer at 77 K; it did not differ much from the value given in [55]. Correlation times of probe rotation τ in the region of fast rotations (5 × 10^–11^ < τ <10^–9^ s) were determined from the EPR spectra by the Equation (1) [56]:

τ = ΔН_+_ × [(I_+_/I_–_)^0.5^ – 1] 6.65 × 10^–10^, [s] (1)

where ΔH_+_ is the width of the spectrum component located in a weak field and I_+_/I_–_ is the ratio of the intensities of the components in weak and strong fields, respectively. The experimental error of τ measured is within ±5% for pristine polymers and ±7% for their blends.

### 2.6. Barrier Properties Evaluation

Permeability tests were performed by a manometric method with a Permeance Testing Device, type GDP-C (Brugger Feinmechanik GmbH, Munich, Germany), according to ASTM 1434-82 (Standard test Method for Determining Gas Permeability Characteristics of Plastic Film and Sheeting), DIN 53 536 in accordance with ISO/DIS 15105-1, and according to Gas Permeability Testing Manual (Registergericht München HRB 77020, Brugger Feinmechanik GmbH, Munich, Germany). Method A was employed in the analysis, as described in the Brugger manual, with evacuation of both top and bottom chambers.

The film sample with a surface area of 0.785 cm^2^ was placed between the two analyses chambers, as previously described by Siracusa and Ingrao [57]. A film mask of aluminum was used to cover the rest of the permeation chamber. The amount of gas flowing through the membrane was determined from the pressure variation due to the gas accumulation in the closed downstream chamber. Gas Transmission Rate (GTR, in *cm^3^/cm^2^ d bar*) was determined considering the pressure increase in relation to the time and the volume of the device. Time lag (t_L_, *sec*), diffusion coefficient (D, in *cm^2^/sec*), and solubility (S, in *cm^3^/cm^3^ bar*) of the test gases were also measured. The mathematical relations used for the calculations were those already reported in the literature [58,59,60,61]. Measurements were carried out at 8, 15, and 23 °C with a gas stream of 100 cm^3^ min^−1^, 0% of gas RH. Chamber and sample temperature were controlled by an external thermostat, KAAKE-Circulator DC10-K15 type (Thermoscientific, Selangor Darul Ehsan, Malaysia). O_2_ and CO_2_ 100% pure food grade gases were used, and all experiments were run in triplicate; results were provided as the average ± standard deviation.

### 2.7. Thickness Determination

The film thickness was determined by using the Sample Thickness Tester DM-G with a digital dial indicator (model MarCartor 1086 type, Mahr GmbH, Esslingen, Germany) with associated DM-G software. The reading was made twice per second, with a resolution of 0.001 mm. The minimum, maximum, and average of each reading was recorded in triplicates, in 10 different positions of each film, at room temperature and reported as mean thickness value, expressed in microns. 

### 2.8. Colour Characterization

The color and transparency of samples were measured using a HunterLab ColorFlex EZ 45/0° TM (mod. A60-1010-615) color spectrophotometer (Hunterlab, Reston, VA, USA) with D65 illuminant, 10° observer, according to ASTM E308 (*Practice For Computing The Colors Of Objects By Using The CIE System*). Measurements were made using CIE Lab scale. The instrument was calibrated with a black and white tile before the measurements. Results were expressed as L* (lightness), a* (red/green) and b* (yellow/blue) parameters. The total color difference (ΔE) was calculated using the following Equation (2):

ΔE = [(ΔL)^2^ + (Δa)^2^ + (Δb)^2^]^0.5^ (2)

where ΔL, Δa, and Δb are the differentials between a sample color parameter (L*, a*, b*) and the color parameter of a standard white plate used as the film background (L’= 66.52, a’= -0.71, b’= 1.16). Chromaticity (C*) and hue angle (h_ab_) were calculated in accordance to the following Equation (3), as previously reported [62,63,64]:

C* = [(a*)^2^ + (b*)^2^]^0.5^ , h_ab_ = tan^-1^(b*/a*) (3)

Measurements were carried out in triplicate at random positions over the film surface. Average values are reported.

## 3. Results and Discussion

### 3.1. Scanning Electron Microscopy 

**Figure 1:**
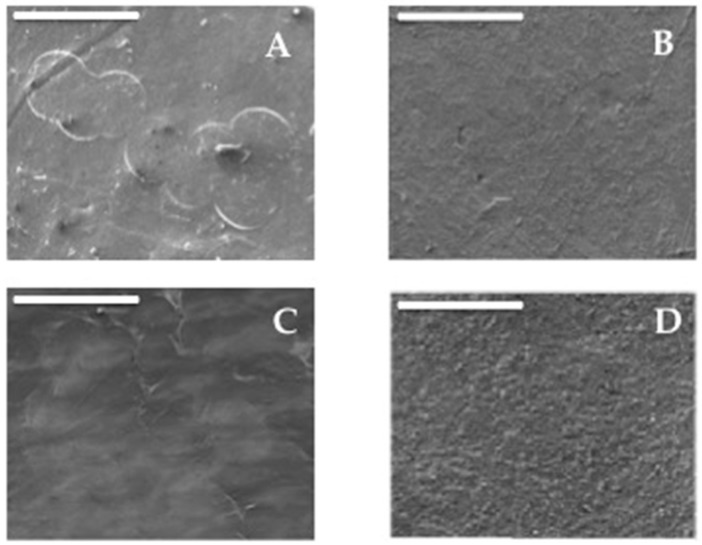
SEM images of Poly(3-hydroxybutyrate) (PHB)/polylactides (PLA) blend surfaces for the films with the biopolymer ratios: (**A**) (1/0), (**B**) (3/7), (**C**) (5/5), and (**D**) (0/1). Scale bar for all micrographs is of 100 µm.

SEM micrographs of the surfaces for the homopolymer films PHB (A) and PLA (D), as well as for PHB/PLA blend films (B, C), recorded in order to evaluate the surface homogeneity and structure, are illustrated in Figure 1 (micrograph of PHB/PLA 1/9 sample was omitted due to poor quality image).

Under given magnification, it could be seen that the surface of homopolymers PHB and PLA displayed, respectively, the textured patterns with coalesced globular elements and finely dispersed entities, forming the rough plane surface. At micrometric scale, micrographs of the blend specimens look like relatively smooth. However, for them the poor relief with the PHB traces of the slightly globular (B) and fibrillar (C) constituents appeared after blending. It is worth noting that the radius of the globules in blend microphotographs (C) was approximately close to 40 μm, which corresponds to the associates of native cell globules of PHB presented in Figure 1A.

Both globular and fibrillar structures could testify the poor blending of two polyesters and partly heterogeneous character of the films’ surfaces. At the same time, as a consequence of PLA content growth in the films, the transition of globular structures (B) to fibrillar ones (C) occurred, which may be connected with partial interaction between the PHB macromolecules and the PLA ones. The morphology structure was supported by further characterization, such as thermal and gas barrier properties, pointing out that a different structural arrangement of the components in the blend could affect the final behavior. 

### 3.2. Thermal Characterization

**Figure 2:**
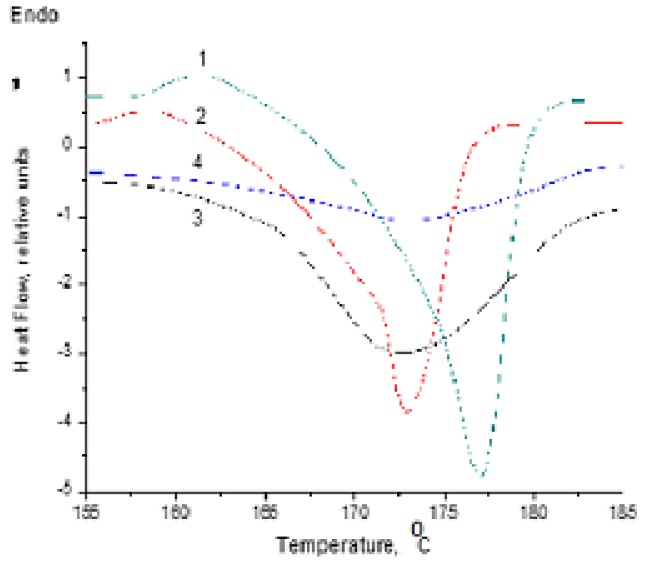
The DSC endotherms in the area of melting for the homopolymers PHB (1) and PLA (2) as well as their blends with the ratio PHB/PLA 5/5 (3) and 3/7 (4) (no data were recorded for sample 1/9 due to poor reproducibility).

The micro-inhomogeneity of PHB/PLA blends brought the structural evolution, with consequent variation in crystalline structure and thermal characteristics. To examine the thermal features, such as melting temperature (T_m_, °C) and enthalpy of fusion (∆H_m_, J/g), in response to crystalline structure changing, DSC technique was applied. Figure 2 reports the DSC heating curves for homopolymers and for PHB/PLA blend films in the area of melting, while Table 1 reports the data in a wider temperature range (20–185 °C). 

Figure 2 and data reported in Table 1 reflect the basic thermophysical transition of the constitutive polymer components in the PHB/PLA films such as crystalline phase melting temperature (T_m_) with corresponding specific melting enthalpy (ΔH_m_), glassy state transition temperature (T_g_), and cold crystallization (T_cc_) with specific cold crystallization enthalpy (ΔH_cc_). As can be observed, biodegradable polyesters PHB and PLA differed in thermal behavior expressed by DSC nonisothermal thermograms. Both biopolyesters had relatively close T_m_ values such as 177 and 172.5 °C for PHB and PLA, respectively. For their blends, the single broadened peak of melting was observed as well, but as the PLA content grew, it shifted from 177 to 173°C, practically to the melting temperature of the PLA homopolymer. The respective values of ΔH_m_ for the 3/7 and 5/5 blends presented the integral thermal effect of both polyesters with an initial crystallinity degree of 64% (PHB) and 51% (PLA), respectively. Therefore, at the lack of melting peaks resolution on the thermograms of the blends, we were not able to calculate the direct contribution of each component to the total endothermic process. In this case, we calculated the crystallinity degree of the PHB/PLA blends (X_c_) according to the Equation (4):

(4) $$X_{c}=\frac{∆H_{m}^{\prime }-∆H_{cc}}{∆H_{m}\left(0\right)W_{\left(PHB\right)}}$$

where ΔH’_m_ is apparent heat of fusion, ΔH_cc_ is the enthalpy of second crystallinity, ΔH_m_(0) is crystallization enthalpy of pure PHB crystal (100% of crystallinity), and W_(PHB)_ expresses the PHB content (the PHB/PLA weight ratio).

The decrease in ΔН_m_ values below the values of the homopolymers could attest to their deviation from the additivity principle, indicating the intermolecular interaction for the polyesters.

Because the T_m_ for PHB slightly exceeded the same characteristic for PLA, the melting of the latter occurred in the solid medium formed by PHB molecules when the segmental interactions between both biopolyesters molecules impeded dynamically. Along with that, for the PLA melted at the lower temperature (173 °C), its crystalline phase fusion could be essentially hindered by the rigid molecular carcass of PHB that is reflected in a sharp drop of total specific enthalpy (see the column in Table 1). Additionally, it is worth noting that authors [65] have recently reported that at temperatures below 150 °C, the PLA could exist in the metastable crystalline state designated as the α’-form with specific enthalpy equals to 107 J/g. This value was used for PLA crystallinity degree calculation.

The cold crystallization temperatures observed for the PHB/PLA blends only had practically the same values and belonged to the PHB predominantly as to the polymer with the slower rate of crystallization [66].

Based on the DSC technique, the immiscibility degree of the polymers in the binary blend can be estimated on the basis of differences in their glass transition temperatures (T_g_) [67]_._ The veracity of such approach is that the higher it is, the greater the T_g_ difference of the homopolymers. By contrast, in the event of complete miscibility, only T_g_ should be observed, and for immiscible blends two specific T_g_ belonging to each of the biopolyesters must exist [68,69]. For PHB and PLA, the constant glass transition was observed, which shifted by −4 °C relative to T_g_ of PLA (see Table 1). The decrease of T_g_ and the constancy of its values in the content interval 0.7–0.5 leave open the question of interaction between PHB and PLA molecules. The ability of such interaction will be considered further in the section devoted to segmental dynamics of the biopolyesters investigated by the probe ESR method.

### 3.3. ESR Spectral Characterization of PHB/PLA Films 

Over two recent decades, temporal (dynamic) and spatial (structural) heterogeneity of biopolymers has been coherently investigated by modern spectroscopic methods and particularly by the probe-radical ESR technique [70,71,72]. The insight into the correlation between blend biopolymer structure and its dynamic molecular behavior is the key step to the design of functional materials for active packaging, biomedicine applications including drug delivery, and environmental problem solution. The previous sections have introduced morphological and thermophysical patterns revealing some specific characteristics of PHB/PLA blend films derived from binary polymer solutions in chloroform particularly. The two succeeding sections will report segmental dynamics of two polyesters that were evaluated by radical-probe ESR technique and gas permeability-diffusion features as principal characteristics of barrier materials for packaging. Atmosphere gas transport in the combination with PHB/PLA segmental mobility indicated by the TEMPO probe rotational diffusion opens the way of creation of environmentally friendly biodegradable material packaging.

**Figure 3:**
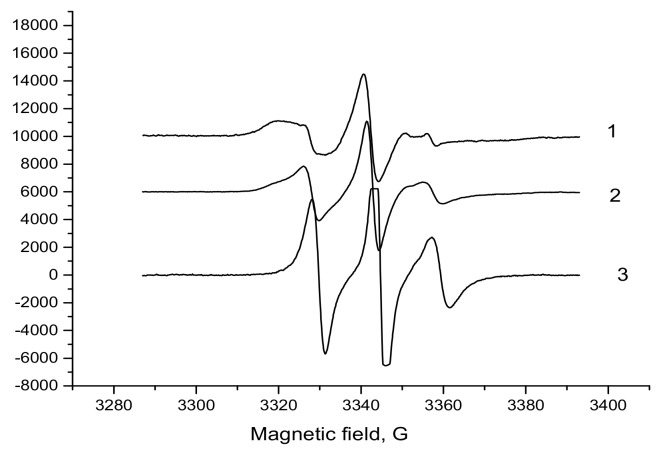
Electron spin resonance (ESR) spectra of PHB, PLA, and PHB/PLA blend films at different polymer ratio: 1 - 1/0, 2 -1/1, and 3 - 0/1.

The ESR spectra of TEMPO-radicals loaded in the PHB/PLA films are represented in Figure 3 for PLA, PHB homopolymers, and their 5/5 blend. 

As in case of the PHB/PLA nanofibers [73], the analogous films are inherently described by the superposition of two single spectra reflecting two different modes of radical rotation with the characteristic correlation times, τ_1_ and τ_2_. Here, the intrinsic correlation time, τ_1_, designates the status of the radical in the denser amorphous fields with slow rotation mobility whilst τ_2_ does the fast, radical rotation in the less dense amorphous fields of the PHB/PLA films. The existence of two TEMPO populations in the amorphous phase of PHB and PLA with distinctive rotation frequencies indicates heterogeneous structure of the intercrystalline area in the biopolymer films. The state of the intercrystalline quasi-amorphous area could be approximated by bimodal model that was earlier proposed for several semicrystalline polymers such as PHB, PLA, and (PET) [74,75,76] and was confirmed for the PHB/PLA electrospun fibers [73]. 

**Figure 4:**
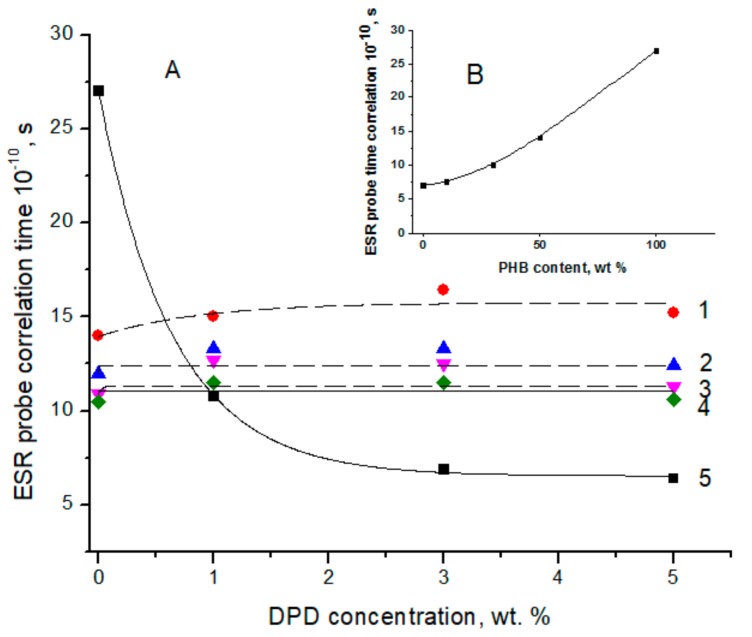
(**A**) dependence of correlation time (τ_C_) on the concentration of active component at the different ratio of biopolyesters PHB/PLA: 0/1 (1), 1/9 (2), 3/7 (3), 5/5 (4), and 1/0 (5). (**B**) dependence of τ_C_ on the PHB content in the pristine blend films without active component.

Figure 4 reports the dependence of correlation time (τ_C_) versus the concentration of active component dipyridamole (DPD) used as modulator in segmental mobility (A) and in respect to PHB content, without DPD content (B).

**Figure 5:**
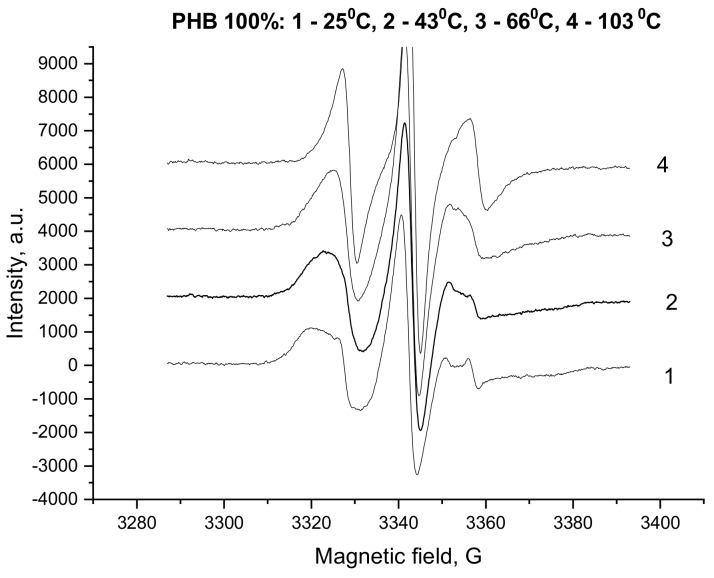
Temperature dependence of the ESR spectra for probe TEMPO in PHB films.

In contrast to the PHB/PLA electrospun fibers, the dependence of correlation time on the PHB/PLA content ratio in the corresponding films has no explicit minimum (see Figure 5 in reference [77]) and monotonically increased (the probe rate of oscillation decreases) with PHB content rise. The minimal and maximal τ_C_ values were observed for homopolymers PHB and PLA, but the intermediate values of τ_C_ belong to their blends in the sequence τ_C_(1/9) > τ_C_(3/7)>τ_C_(5/5) where, in parentheses, the PHB/PLA fractions are given. In Figure 4A, the largest span in the probe mobility complies with the PHB films containing the low-molecular additive, dipyridamole (DPD), at concentrations in the range from 0 to 5 wt.% as modulator segmental mobility. Actually, when the TEMPO-probe could not penetrate the crystalline entities due to steric hindrances, the increase in the concentration of the high-crystalline PHB should decrease an averaged distance among dispersed crystallites and, hence, enhance the restrictive impact on segmental dynamics of intercrystalline macromolecules. The availability of the third component (DPD) in the polymer films affected the mobility of the probe in a complex way, and dramatically decreased the range of the τ_C_ values for PHB as maximally crystallized biopolymer and weakly influenced the τ_C_-concentration dependence. The maximal crystallinity degree of PHB led to the minimal fraction of amorphous (intercrystalline) volume. The PLA molecules, being embedded there, disturbed the conformation of PHB molecules and because of interaction with polyester functional groups, confined segmental dynamics as well. For the blends with relatively low values of ΔH_m_ and therefore with low crystalline degree (see ΔH_m_ values in Table 2), the amorphous space among the crystallites was enlarged. Therefore, the probability of intermolecular interaction between the pair of ester groups for PHB and PLA became essentially lower, and in the absence of such interactions, the segmental mobility was higher.

In Figure 5, the temperature variation of probe ESR spectra for PHB is presented in the range 25–105 °C. The temperature span was chosen to exclude the glassy-state transition that typically occurs between 5 and 20 °C and thereby avoid the change in the mechanism of probe rotation from the “slow” in the glassy state to the “fast” characterizing PHB elastic state.

With the temperature increase, the broad spectra changed into sharper ones with the narrower peak separation. This effect indicates that the spin probes begin to rotate with higher rate due to the growth of segmental mobility. For the obtained ESR spectra, the correlation time τ_c_ was estimated using the conventional formula, which is reasonable for the specimens above glassy state temperature, (see Equation (1) reported in the experimental section).

**Figure 6:**
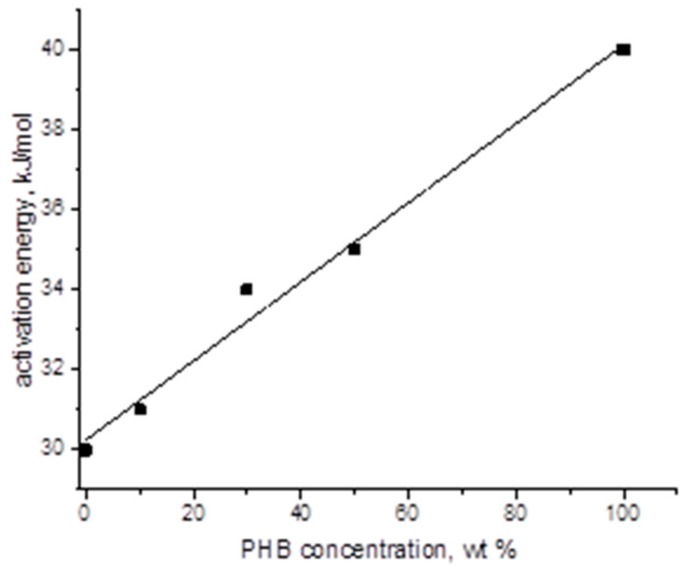
Energy activation of TEMPO probe rotation in PHB/PLA blends.

Figure 6 summarizes the effective activation energies for the rotational correlation times (E_τ_) that were obtained via the fitting in an Arrhenius semilogarithmic coordinates, analogously to the previous procedure performed for pristine PLA [31] and PHB [53]. 

In these works, it has been shown that there are two different segmental dynamic regimes: (a) At the high-temperature range T > T_g_, where the values of ln(τ_c_) [s/rad] decreased quickly with reciprocal temperature (in K^−1^); and (b) at the low-temperature range T < T_g_. For the PHB/PLA blends given, however, no dramatic change in mobility and activation energy was observed in the range of T_g_ values between 39 and 43.5 °C. The monotonically increasing values of E_τc_ with the PHB content demonstrate the enhancing hindrance to diffusion of radical moving because of the crystallinity growth in the blends.

Earlier, a number of comprehensive works [70,78,79,80] were devoted to polymer micro heterogeneity exploration by probe ESR technique. In particular, this phenomenon was carefully investigated for PLA as a potential packaging material [24]. The pioneer papers [68,70] stated that the dimension of structural inhomogeneity may differ depending on which method is used for its evaluation. If the custom DSC data for T_g_ measuring are based on micro-spatial averaging in a polymer volume, the averaged dimension of heterogeneity in the same object are comparative to the size of the probe. For the TEMPO, it should take about 1nm [70]. To avoid this discrepancy between the quasi-thermodynamic and dynamic approach, Kusumoto and collaborators [81] and Bullock, Cameron, and Miles [82] introduced the special correlation characteristic T_5mT_ that is physically determined in the framework of free volume theory of Bueche [83]. In our subsequent planned works, we will try to apply this fruitful approach for the consideration of dynamic behavior at essentially lower temperatures (T < T_g_) to connect thermal and dynamical data of the blends under investigation. 

### 3.4. Gas permeability Characteristics

#### 3.4.1. Isothermal Permeability

Gas transport in biodegradable plastic packaging is subject to the penetrant chemical structure, external conditions such as pressure, temperature, humidity, etc., as well as the polymer features including crystallinity, morphology, heterogeneity, the pores’ architecture, and others. 

**Figure 7:**
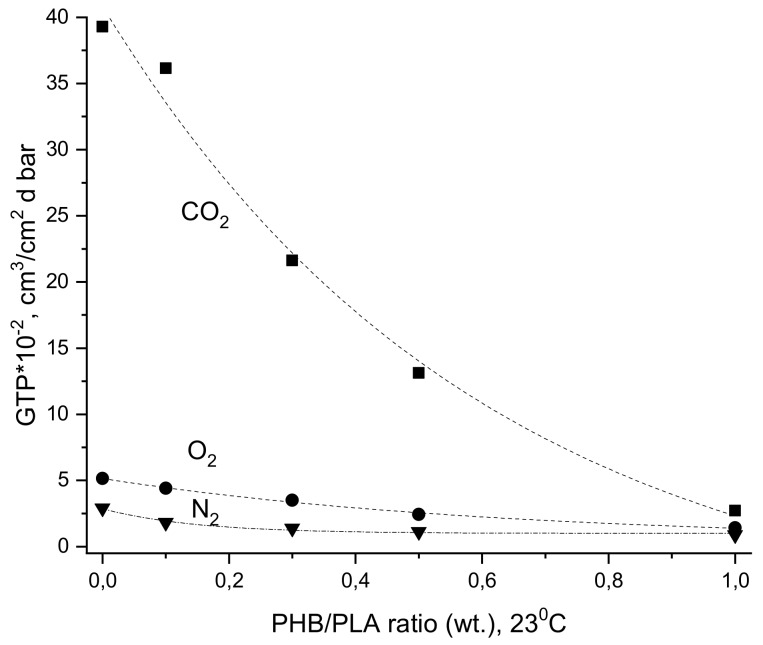
Gas transport characteristic dependences on ratio of PHB/PLA film sample, at 23°C.

The measurement results for transmission of basic atmospheric gases such as N_2_, O_2_, and CO_2_ through PHB, PLA, and their blends with various contents are presented in Figure 7.

As seen for all compositions, the gas transmission rates (GTR) for these gases correspond to the following sequence

GTR(CO_2_) > GTR(O_2_) > GTR(N_2_)

The transmission of all gases through pristine PHB was lower than that for pristine PLA. 

It is well recognized that at the molecular level, gas transmission is driven by gas solubility and diffusion, and the gaseous permeability coefficient represents the product of diffusivity and solubility coefficient [84,85,86]. Gas diffusional transport and utmost sorption capacity (thermodynamic solubility) depend on polymer segmental mobility and thermodynamic affinity of functional groups for gas molecules, respectively. Further, the fraction of crystallinity and the morphology of crystals have a big impact on gas diffusion. Particularly, during gas transport, the gas molecules have to increase a diffusion pass trajectory, tortuosity, to move around impervious objects in the form of polymer crystalline entities. At high degree of crystallinity, which is representative for PHB, the distance between any pair of crystals (crystallites or lamella) is critically reduced, leading to a segmental mobility decrease of PHB transition chains situated in the intercrystalline area accessible for gas transmission.

As glass transition temperatures (T_g_) for PHB and PLA vary by several tens of degrees, namely ≈ 20 °C and ≈ 60 °C, respectively, at room temperature, each of these biopolymers is in a different physical state. Namely, PHB with T_g_ that just below ambient temperature displays viscoelastic behavior limited by high crystallinity while PLA is rather characterized by glassy-state features. Restricted molecular mobility for both polymers should reflect failure of both mechanical and transport characteristics.

**Figure 8:**
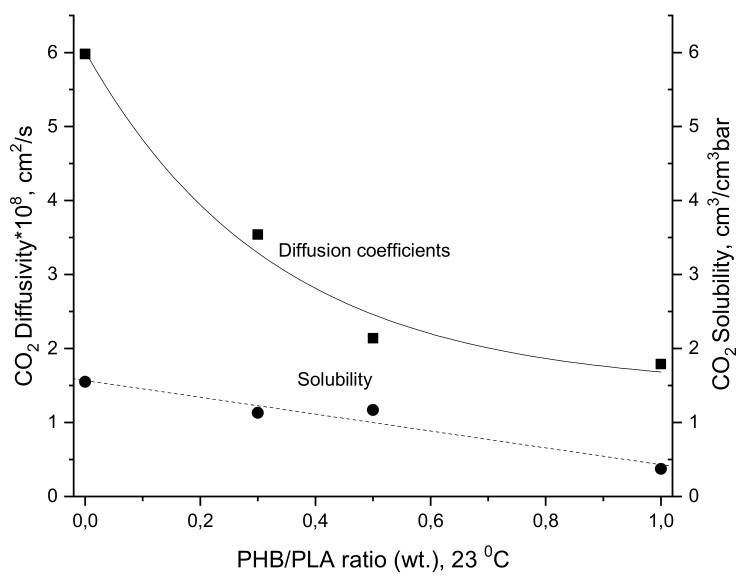
Diffusivity and solubility dependences versus PHB/PLA ratio.

For the high content of PLA, Figure 7 shows the selectivity of the gas pairs CO_2_/N_2_ and CO_2_/O_2_, which were much higher than those for PHB. As it was noted in our previous works [31,53], CO_2_ has the highest polarity and its affinity to the polar ester groups has to be the biggest one as compared with O_2_ and N_2_. For all PHB/PLA compositions, the CO_2_ gas transmission essentially exceeds the analogous characteristics of O_2_ and N_2_. The significant difference in the CO_2_ transport characteristics for pristine polymers PHB and PLA is due to the inequality in ester group concentration accessed to the penetrant molecules’ interaction. For PLA with relatively low crystallinity, this parameter was essentially larger than for PHB owing to the higher ester concentration, more specifically due to the higher ratio of ester/hydrocarbon groups. In pristine PLA, the O_2_/N_2_ perm-selective ratio was larger when compared to all other samples. Since O_2_ is a molecule with smaller critical volume in comparison to N_2_, it can more easily diffuse by activation mechanism. The main idea of the influence of PHB/PLA ratio on CO_2_ diffusivity and solubility is reflected in Figure 8 where the decrease of two fundamental characteristics (D and S) is clearly revealing.

In contrast to P and D, the gas-penetrant solubility coefficient (S) is a thermodynamic characteristic that is determined by penetrant condensability as well as by intensity of biopolymer–penetrant interaction. Its values are in the same order of CO_2_ > O_2_ > N_2_. The analogous trend in CO_2_ sorption in PHB/PLA blend films is in good conformity with earlier published papers [87,88,89]. The good accordance is found to be related to the critical temperature of the gas penetrants, whereby CO_2_ with a critical temperature of 304.15 K is highly condensable within the polymeric matrix in comparison to oxygen (154.55 K) and, subsequently, nitrogen (126.2 K) [90,91]. Solubility always favors those of higher critical temperature since it indicates tendency in gas penetrants to condense within the polymer.

#### 3.4.2. Temperature Dependency of Gas Permeability

**Figure 9:**
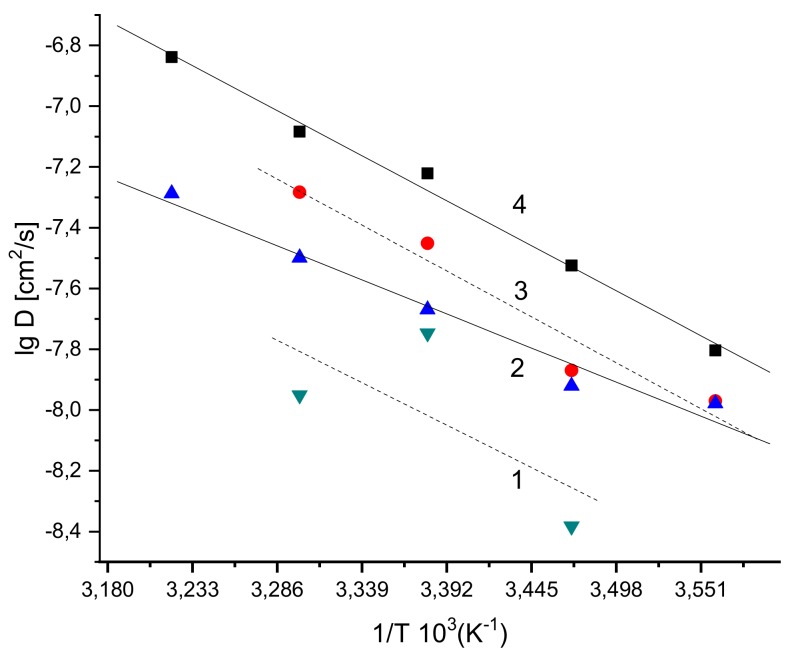
CO_2_ diffusivity in PHB/PLA blend films as the function of reciprocal absolute temperature. PHB/PLA ratios are: (1) PHB; (2) PHB/PLA 5/5; (3) PHB/PLA 3/7; (4) PLA.

On the way to implementing food packaging, the permeability dependency on temperature is a major subject that should be taken into consideration for the food safety control. In concordance with our above data in Section 2 and Section 3, the temperature interval given in Figure 9 does not include thermal transition points for both biopolyesters, PHB and PLA, and as it follows from this figure, the CO_2_ transport through blend polymer films satisfies the Arrhenius Equation (5) in conventional semilogarithmic coordinates:

(5) $$P=P_{0}\times exp\left(-\frac{E_{p}}{RT}\right)$$

where P_0_, E_p_, and R are the pre-exponential factor of permeation related to transport entropy, the transport activation energy, and the gas constant, respectively.

The analogous expression could be written for the energy activation of diffusion (Equation (6)):

(6) $$D=D_{0}\times exp\left(-E_{d}/RT\right)$$

where D_o_ and E_d_ are the pre-exponential factor of diffusivity and the diffusion activation energy, respectively. 

The temperature dependence of thermodynamic solubility, ΔHs, of carbon dioxide in the PHB/PLA blend matrices is described by the Van’t Hoff expression that was presented in Equation (7):

(7) $$S=S_{0}\times exp{\left(-\frac{∆H_{s}}{RT}\right)}_{}$$

where S_0_ is a pre-exponential factor and ΔH_s_ is the apparent molar heat of equilibrium gas sorption.

The comparison of rotation diffusivity energy activation for the radical probe TEMPO (32–42 kJ/mol) (see Figure 6), with CO_2_ permeation energy activation for molecules (40–53 kJ/mol) in PHB/PLA reported in Table 2 shows that both characteristics are close enough. In the given temperature range, consequently, the primary mechanism of CO_2_ activated transport is segmental mobility of the blended biomacromolecules. With decreasing the permeability temperature and approaching the glass transition point, the enhancement in the discrepancy increment for E_τ_ and E_D_ could be related to the exchange of segmental motion mechanism, namely the predominance of the pendant group mobility in comparison with segmental translation.

### 3.5. Color Evaluation

When polymer materials are used for food packaging application, the film transparency and color are important requisites. In order to evaluate the food quality, color is one of the key factors evaluated, especially from consumers, and is considered as one of the primary criteria affecting food appearance, taste, and freshness [92]. In general, in order to attract the consumers’ attention, packaging has to interfere as little as possible with the food color. Our film surface color results are reported in Table 3, for PHB-PLA samples, compared to white standard.

All films appeared clear, showing a slightly different transparency, despite their different crystallinity. In order to explain the results, as previously reported by Siracusa et al. and by R.G. McGuire [53,92] the CIE Lab Color scale was considered. In the scale, the lightness coefficient (L*) ranges from black (0) to white (100) and, for any L* value, a* and b* values allocate the color on a rectangular coordinate grid, perpendicular to the L* axis. At the axis origin (a* = 0 and b* = 0), the color is achromatic (grey). Considering the horizontal axis, a positive a* value indicates a hue of red-purple and a negative a* value indicates a green hue. Considering the vertical axis, a positive b* value indicates a yellow hue and a negative b* value indicates a blue hue. All films showed similar L* value, showing the same translucency, whereas a* and b* indicated a faint tendency toward a yellowish color (*h*_ab_ over 90°). The low C* values recorded means low color saturation and, consequently, a good transparence of the films, despite the different copolymer compositions.

## 4. Conclusions

The transformation from custom packaging materials based on petrochemical non-biodegradable polymers to bio-based biodegradable ones as the innovative gas barriers with selective permeability encourages intensive scientific and industrial explorations. Here, the multifaceted approach combining dynamic (gas permeation and probe-radical ESR technique) and structural (SEM, DSC, and color estimation) methods is given to describe the complex behavior of fully bio-based blends based on two polyesters: poly (3-hydroxybutyrate) (PHB) and polylactic acid (PLA). Both polymers were considered as the real competitors that have to replace petrol polymers in the packaging industry. 

Both probe rotation mobility and atmospheric gas permeation as well as their diffusivity monotonically decreased during the transition from PLA to PHB for all component ratios, namely because of transferring from PLA with lower crystallinity to PHB with higher one. The impermeable crystalline entities impede gas transport due to diffusion pass extension. The temperature dependences for CO_2_ permeability and diffusivity as well as for probe correlation time allow the authors to evaluate the activation energy of both processes. The values of gas transport energy of activation and the same characteristic for TEMPO rotation mobility are substantially close to each other, which evidently support that polymer molecular mobility determines the gas permeability.

## Figures and Tables

**Table 1:** Differential scanning calorimetry data for homopolymers PHB and PLA and for PHB/PLA blends.

Sample PHB/PLA (w/w)	T_g_ (°C)	T_cc_ (°C)	Tm (°C)	ΔH_cc_ (J/g)	ΔH_m_ (J/g)	Χ_c_* (%)
1/0		-	177		93.0	64
3/7	40.0	69.9	173	2.3	30.7	50
5/5	39.5	67.8	172	2.6	38.2	49
0/1	43.5	-	172.5	-	54.8	51

* Calculated from referenced specific enthalpy of melting: 107 J/g for PLA [65] and 146 J/g for PHB [66].

**Table 2:** Energy activations of CO_2_ permeability (E_P_) and diffusion (E_D_) combined with CO_2_ molar enthalpy of solubility (ΔH_S_) for pristine PHB and PLA and their blends.

PHB/PLA sample (w/w)	E_P_ (kJ/mol)	E_D_ (kJ/mol)	ΔH_S_ (KJ/mol)
1/0	22.8 ± 4.4	50.6 ± 4.5	−27.8 ± 4.5
3/7	41.4 ± 2.4	54.4 ± 4.9	−13.1 ± 3.5
5/5	32.5 ± 1.5	40.6 ± 4.2	−8.1 ± 3.8
0/1	24.9 ± 4.5	53.6 ± 3.0	−28.7 ± 3.7

**Table 3:** Lightness coefficient (L*), a*, b*, total color difference (ΔE), C*, and h_ab_ of PHB-PLA films.

PHB/PLA Sample	L*	a*	b*	ΔE	C*	*h*_ab_
White standard	67.16 ± 0.02	−0.69 ± 0.01	1.25 ± 0.01	-	0.93	138
1/0 (PHB)	34.49 ± 0.67	−0.24 ± 0.03	−1.69 ± 0.13	32.70	1.71	98
1/9	35.60 ± 0.01	−0.43 ± 0.01	−1.10 ± 0.01	31.61	1.18	111
3/7	35.02 ± 0.04	−0.36 ± 0.01	−1.96 ± 0.01	32.24	1.99	100
5/5	34.31 ± 0.02	−0.34 ± 0.02	−1.77 ± 0.01	32.94	1.80	101
0/1 (PLA)	33.66 ± 0.31	−0.67 ± 0.01	−3.14 ± 0.01	33.71	3.21	102

h_ab_ = 0°, red–purple; h_ab_ = 90°, yellow; h_ab_ = 180°, green; h_ab_ = 270°, blu.
